# Habitat Structure, Quality and Landscape Predict Species Richness and Communities of Collembola in Dry Grasslands in Austria

**DOI:** 10.3390/insects9030081

**Published:** 2018-07-07

**Authors:** Pascal Querner, Norbert Milasowszky, Klaus Peter Zulka, Max Abensperg-Traun, Wolfgang Willner, Norbert Sauberer, Christine Jakomini, Thomas Wrbka, Ingrid Schmitzberger, Harald G. Zechmeister

**Affiliations:** 1 Department of Integrative Biology, University of Natural Resources and Applied Life Sciences Institute of Zoology, Gregor-Mendel-Str. 33, A-1180 Vienna, Austria; 2 Department of Integrative Zoology, University of Vienna, Althanstr. 14, A-1090 Vienna, Austria; norbert.milasowszky@univie.ac.at (N.M.); klaus.peter.zulka@univie.ac.at (K.P.Z.); max.traun@me.com (M.A.-T.); 3 Institute of Science in Conservation, University of Applied Arts Vienna, Expositur Salzgries, A-1010 Vienna, Austria; 4 VINCA-Vienna Institute for Nature Conservation and Analyses, Giessergasse 6/7, A-1090 Vienna, Austria; wolfgang.willner@univie.ac.at (W.W.); norbert.sauberer@vinca.at (N.S.); 5 Environment Agency Austria, Spittelauer Lände 5, A-1090 Vienna, Austria; 6 Ministry of Sustainability and Tourism, Division for Nature Conservation and Species Protection Stubenbastei 5, A-1010 Vienna, Austria; 7 Department of Botany and Biodiversity Research, University of Vienna, Rennweg 14, A-1030 Vienna, Austria; pascal.querner@gmx.at (C.J.); thomas.wrbka@univie.ac.at (T.W.); ingrid.schmitzberger@coopnatura.at (I.S.); harald.zechmeister@univie.ac.at (H.G.Z.)

**Keywords:** dry grassland patches, fragmentation, soil mesofauna, dispersal, patch size

## Abstract

We assessed the relationships between site size, habitat quality, landscape factors (fragmentation, landscape diversity) and species richness in communities of Collembola in 50 small dry grassland habitat patches in an agricultural landscape of eastern Austria. Grasslands in that region were once widespread and extensive, but have become increasingly fragmented and isolated. We hypothesized that dry grassland springtails species richness is significantly correlated with site variables (soil properties, habitat quality) and that the size of grassland sites is positively correlated with species richness. We used pitfall traps in 50 dry grasslands in differently structured agricultural landscapes and tested total abundance and three species richness measures: (1) the number of dry grassland specialist species; (2) total number of dry grassland species and (3) overall species richness. In the multivariate correlation models, we found that all species richness measures were significantly related to the plant species richness, a shape parameter of the sites, soil properties such as humus, temperature, sand and gravel content and the landscape variable reflecting isolation (distance to the nearest large dry grassland area). This landscape variable indicates that neighbouring grasslands are influencing the species richness of the sites. This may be a result of passive wind dispersal across the landscape or historic connection of the small sites with much larger dry grasslands. The size of the site did not show any significant correlation with total, dry grassland specialist, dry grassland generalist or generalist species richness. The small size of Collembola might explain these findings, because they have high population densities even in small patches.

## 1. Introduction

Soil is part of most terrestrial ecosystems and supports above-ground biotic patterns and processes. A variety of animals inhabit the soil and contribute significantly to the decomposition of dead organic matter [1], nutrient cycles [2], the development of soil microstructure [3,4] and thus to the functioning of the ecosystem as a whole [5]. In temperate regions, Collembola often form the most abundant arthropod group in the soil (10^4^–10^5^ individuals m^−2^; [2]). They belong to the soil mesofauna (body size of 0.2–2 mm) with a species richness of up to 60 species in a beech forest of 0.5 ha [6]. They live in the soil pores, the litter layer, on the soil surface, and on vegetation [7].

The diversity and community composition of springtails are usually determined by abiotic and biotic site (or patch) parameters, such as soil type, temperature, moisture, acidity, the presence or absence of a litter layer and the fungal community influence communities [8,9,10,11,12,13]. Parameters at the local scale with the highest influence are soil acidity, vegetation type, moisture and humus form [14,15]. However, it is not yet fully understood to what extent these parameters influence the structure and composition of Collembola communities at local scales.

Landscape factors like fragmentation or landscape structure could also affect springtails but have rarely been investigated by soil ecologists [16,17]. The land-use diversity (see [18]) around a patch could be important as even small Collembola can move from one habitat to the other or disperse passively by wind over large distances. In recent years, some studies have investigated the effects of the landscape on springtails species richness and communities [19,20,21,22,23,24,25,26,27,28,29,30]. All these studies indicate that landscape-scale effects can be relevant for Collembola communities. However, the importance of landscape scale effects like fragmentation for small soil animals (0.2–2 mm body size) is still poorly studied. It is not clear how patch-dependent species respond to the fragmentation and isolation of threatened biotopes such as dry grasslands, and to secondary fragmentation implications such as edge effects and a change in habitat quality.

Until a few decades ago (about 50 years), dry grasslands used to cover large areas in Eastern Austria [31,32]. These grasslands were widely used as pastures for sheep grazing and contained a high biodiversity of specialised animal and plant species [33,34,35,36]. Over the last century, large areas have been lost due to land use intensification (for example conversion into vineyards), resulting in many small and strongly fragmented grassland patches (about 80 in the region). Other sites have become overgrown due to shrub and woody plant encroachment in areas no longer mown or grazed [37]. These small dry grassland sites (patches) were selected to study the effects of habitat fragmentation and landscape composition on Collembola. For springtails diversity and their conservation in Austria, dry grasslands are important as they contain a high number of specialised species, with many species restricted to these biotopes [38,39,40,41,42,43,44,45,46,47,48,49].

In this study, we selected 50 of these dry grassland sites and investigated the effects of habitat quality, habitat structure and landscape factors and correlated them with Collembola species richness and communities. We hypothesized that dry grassland springtails species richness is significantly correlated with site variables (soil properties, habitat quality) and that the surrounding landscape is not a relevant factor. We further hypothesized that the size of grassland is positively correlated with total species richness. We categorized all species according to their habitat requirements to better understand the effects on dry grassland dependent species, as they are more important for conservation of dry grassland communities.

## 2. Material and Methods

### 2.1. Study Area and Site Selection

**Figure 1:**
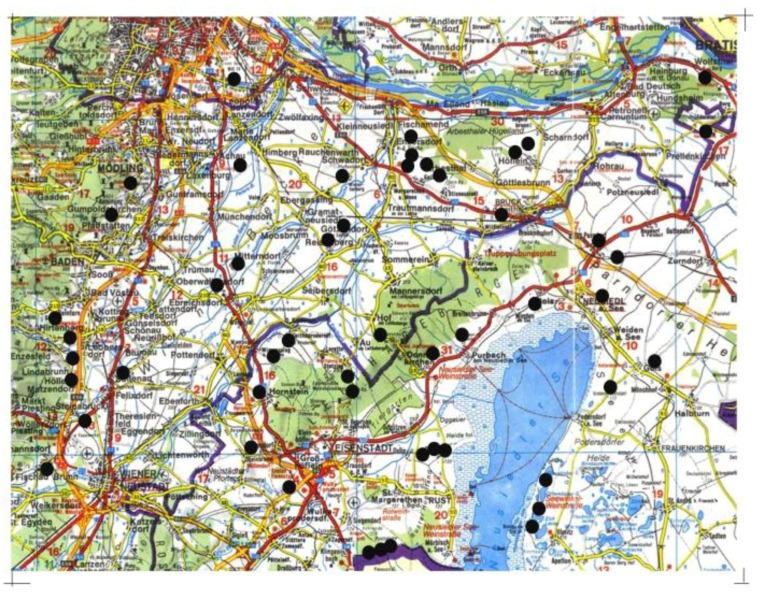
Map of Eastern Austria with the 50 dry grassland sites.

Fifty dry grassland sites were selected in Eastern Austria, south of the river Danube in the Vienna basin (Figure 1). The climate is subcontinental with moderate precipitation (610–660 mm) and average temperatures between 9.4 °C and 9.8 °C and an annual temperature amplitude of about 20 °C between the temperature average of the coldest and the warmest month (Walter and Lieth 1967). The rainfall averaged among the sites during the sample period with a range of 22 mm at Gumpoldskirchen to 7 mm at Eisenstadt and Bruckneudorf. Sites were selected to obtain a large diversity in size, quality and landscape parameters but restricted to dry grasslands of the Festuco-Brometea class [36,50]. The selection was based on two databases on dry grassland sites in the region [32,51]. To avoid effects of spatial autocorrelation, only sites were retained that were situated at least 1 km apart. The areas of the sampled sites varied between 0.01 ha and 10 ha, with an altitudinal range of 117–290 m a.s.l. In order to avoid effects of spatial autocorrelation, only sites at least 1 km apart were selected.

### 2.2. Collembola Sampling

We sampled the epigeal Collembola that actively move on the soil surface and within the litter layer using pitfall traps across all 50 sites. Glass jars of 4.5 cm diameter were half-filled with ethylene glycol (100%) as a preservative and some droplets of detergent. In the centre of each site, three traps were placed 5 m apart in the corners of a triangle. No roofs were used to avoid microclimate modification. Traps were left open for 21 days (from 12 April 2001 to 4 May 2001). Due to logistical constraints, the catch of the three traps at each site was pooled in the field, and analyzed together.

### 2.3. Collembola Determination and Classification

Collembola were identified using the keys of [52,53,54,55,56,57,58]. Each species was classified into one of three categories depending on their degree of habitat specialization. Categorization of species are based on published literature [16,42,43,44,45,46,47,48,49,59] and used the following categories:

Category 1—Dry grassland specialists: Organisms depending on the habitat patches, the agricultural matrix is inhabitable and crossing it is a rare phenomenon. The category comprises species highly dependent on dry grassland patches.

Category 2—Dry grassland species: organisms use and benefit from dry grassland patches, but are not restricted to these habitats. Even if they prefer dry grassland fragments, they also reproduce in other dry habitat types of the landscape. They can use stepping stones and bridges that facilitate movement across the landscape between grassland patches.

Category 3—Generalist species: Organisms which occur in dry grassland patches as well as large parts of the agricultural matrix because of similarity of ecological conditions, in particular openness and dryness. This category includes generalists and species that are able to tolerate high disturbance levels of an agricultural matrix.

As dependent variables, we used total springtails activity abundance, dry grassland specialists species richness (category 1), dry grassland total species richness (category 1 + 2), generalist species richness (category 3) and total species richness.

### 2.4. Patch Variables

The local site variables can be divided into parameters describing soil parameters, habitat structure, habitat quality and historic site area.

1. To obtain data on local habitat quality, we analysed soil texture and soil composition. In each site, we took three soil samples from the 5 × 5 m square in the centre of the site during August 2001. Soil cylinders of 10 cm^2^ surface area and 10 cm depth were homogenised and dried at 105 °C. Dry sieving separated gravel content (>2 mm) from the remaining fine soil (<2 mm). The fine soil components were further separated by sedimentation analysis. Percentages of sand fractions, silt and clay refer to fine soil; the percentage of gravel content refers to total soil weight. Soil humus content was determined by burning fine soil at 500 °C and assessing the weight loss (done by the Austrian Federal Agency for Soil Analysis). We determined soil temperature sums during a 25-day period in August and September 2001 using the polarimetric sugar inversion method of [60]. In each site, two plastic tubes containing 20 mL of sugar solution were buried in 5 cm depth. During transport, the samples were kept in a cold box. Rotation angles were measured immediately before the start and shortly after the end of the exposition period with a circular polarimeter (Atago Polax-D). We used rotation angles instead of mean temperatures in the analysis.

2. Habitat structure: the descriptors site area and geometry (mean parameter area ratio—MPAR) and landscape composition around the site (AGMT, SHADE) were measured. These variables were obtained from aerial photographs using the programs ArcView Patch Analyst (ESRI Inc., Redlands, CA, USA), GIS-techniques and ground-truthing (field validation of the information’s from the areal photographs).

3. Habitat quality: The vegetation structure of the site (grass and shrub area) was obtained by ground-truthing. Additionally, we measured vegetation density in the sites in April and August 2001 using a pasture disc meter [61,62]. A 20 × 20 cm disc of 100 g of weight was released from 1 m height to its final position above the ground was measured. Measures were made within each 20 × 20 m plot in the centre of the site to calculate the average cover.

We also sampled vascular plants within a 5 × 5 m plot in the centre of the site (see [36] for detail sampling design). All species were recorded, and their quantity was estimated using a modified Braun-Blanquet scale [63]. Bryophytes were collected once in April 2001, a time of the year known to be optimal for bryophyte growth in the investigated area, e.g., [64]. Thirty minutes were spent recording all bryophytes within a 5 × 5 m square in the centre of the site. Specimens were collected and identified in the laboratory. Vascular plants and bryophytes were classified in a similar way as the Collembola and the species richness used for the analysis (total plant species richness, strictly dry grassland plant species richness (category 1), total bryophytes species richness, strictly dry grassland bryophytes species richness (category 1).

4. Historic site size: To compare current and historical site areas, aerial photographs from 1950 to 2000 were scanned and the historical site area and the current site area were delineated. This shows how much site area was lost in the last 50 years.

### 2.5. Landscape Variables

The landscape variables can be divided into variables describing fragmentation and land use intensity of the surrounding landscape. These variables were obtained from aerial photographs using the programs ArcView Patch Analyst (ESRI Inc.), GIS-techniques and ground-truthing. Within 1 × 1 km squares (with the site in the centre of this square), spatial elements were delineated to one of 62 pre-defined land use types and 10 pre-defined land cover classes were assigned to each landscape element.

1. Fragmentation: To assess the influence of large dry grasslands in the proximity of the sites, the distance between site centre and the nearest large dry grassland area (>15 ha in size) was measured on a topographical map (scale 1:50,000).
2. Land use intensity of the surrounding landscape: To obtain a simple measure for ‘landscape heterogeneity’ the number of land use types in the 1 × 1 km^2^ quadrat around the site was counted. Five combinations of element types and attributes were specified, (a) dry grassland elements; (b) elements with extensive agricultural use; (c) fallow-dominated elements; (d) short-grassed elements; and (e) linear elements of various kinds. The summed area within a 1 × 1 km^2^ quadrat area around each patch was calculated and used as a predictor for species richness in the patches. To quantify land use intensity the 62 pre-defined land use types in the 1 × 1 km^2^ quadrat around the patch were classified as high, low or neutral land use intensity. Natural forests or other grasslands are considered to reflect low land use intensity. In contrast, intensively used agricultural fields are associated with high land use intensity. An average land use intensity weighted by area was calculated to obtain a factor describing land use intensity of the landscape around each patch.

### 2.6. Statistical Analysis

In the first step, we performed a Canonical Correspondence Analysis (CCA) to ordinate sites and to analyse which site or landscape variable separates Collembola assemblage of the 50 sites. Only presence/absence data were used for the ordination of the communities, as abundances collected with pitfall traps are activity based. Canoco for Windows version 4.0 (Biometris–Plant Research International, Wageningen, The Netherlands) was used for the CCA ordination [65].

In a second step, Pearson correlations were calculated with SPSS Statistics for Windows, version 10.0 (SPSS Inc., Chicago, IL, USA) to determine all significant variables for the total abundance and the different species richness numbers (dry grassland specialist species richness (category 1), dry grassland species richness (category 1 + 2), generalist species richness (category 3) and total species richness.

We then calculated regression models for the species richness numbers using the multivariate regression procedure in SPSS 10.0 with backward elimination using all plant species richness, site area 1950, site area 2000, patch shape (MPAR), patch total shrub area, patch vegetation variation, patch vegetation cover, soil properties, soil temperature, percentage of bordering agricultural land (MT Agric), habitat management (H-MGMT), distance to the nearest large grassland area and total dry grassland area in 1 × 1 km.

## 3. Results

A total of 56,000 individuals were captured and determined to species level; 86 different species were found in total, six species were new to the Austrian fauna [49]. All species and their category are listed in Table 1. Nine species were considered dry grassland specialist species (category 1) and ten species belong to dry grassland generalist species (category 2). The total species richness varied between 13 and 24 per patch. The dependent variables for all patches are listed in Table 2.

### 3.1. CCA

**Figure 2:**
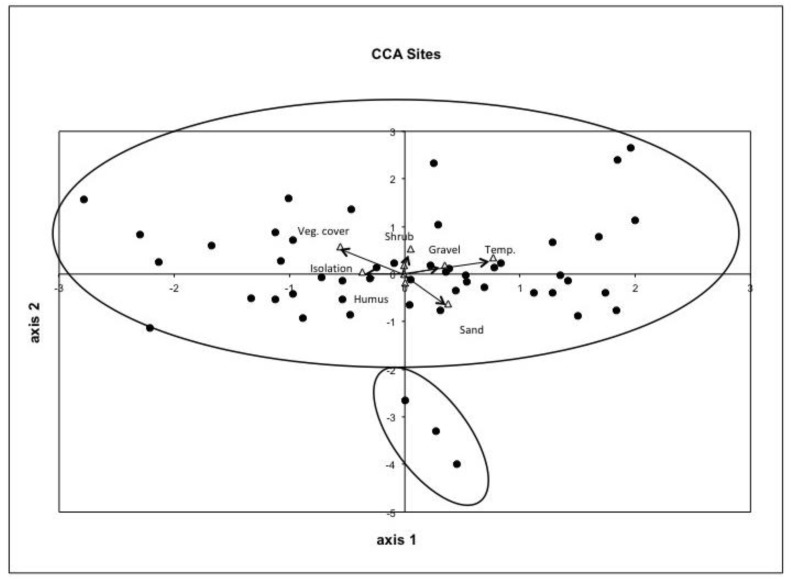
CCA ordination of the surface active Collembola collected with pitfall traps in 50 dry grassland sites in Eastern Austria. Four clusters and the patch and landscape variables separating the patches and clusters are shown.

The CCA ordination of the patches shows the separation of the Collembola communities according to the variables vegetation cover, shrub overgrowth, isolation, sand and gravel content and soil temperature represent the most important variables (Figure 2). Sites 37, 38 and 39 can be separated from the main cluster on the axis 1. They are all found on the south-eastern border of Lake Neusiedel (see Figure 1).

Most sites are spread along axis 1. On the left side sites 06, 08, 12, 25 and 43 are characterised by high vegetation cover, low soil temperature and long distance to a mainland. Their soils have low amounts of sand and gravel. These sites contained a low number of springtails species of category 1 and 2, but a high number of generalist species. On the right side (01, 02, 03, 05, 14, 17, 21, 23, 27, 40 and 49) are separated mainly by high soil temperature, sand and gravel content and a low vegetation cover, they have a high number of category 1 and 2 species and represent sites of high quality for Collembola. In the centre is a group of sites that are not influenced by extreme environmental conditions and form the largest part of the investigated grasslands (66%). All 47 sites of the main cluster can not clearly be separated from each other but show a gradient from left to right with vegetation cover and isolation on one hand and temperature, sand and gravel content on the other hand as important factors for this gradient.

### 3.2. Single Correlations

No single significant correlation was found with total abundance, total species richness and the species richness of category 1 (dry grassland specialists). All significant correlations for the dry grassland specialist species richness and generalist species richness are listed in Table 3. For the dry grassland specialist species richness of Collembola (category 1), site variables dry grassland plants species richness, soil gravel content and soil temperature were positively correlated and the landscape factor distance to the nearest large grassland (mainland; a potential source area) was negatively correlated. Generalist springtails species richness was positively correlated with total plant species richness and negatively correlated with total dry grassland area in 1 × 1 km.

### 3.3. Multivariate Regression Models

Backward selection multivariate regression modelling revealed total plant species richness, shape of the grassland (MPAR) and soil temperature as significant predictors of dry grassland specialist species richness and also the dry grassland total species richness. For dry grassland specialist species richness, the landscape factor distance to the nearest large dry grassland area was also found to be significant in the model.

For generalist species richness, the patch factors total plant species richness and humus content in the soil emerged as significant predictors in multivariate regression modelling.

Total species richness is efficiently described with the variable shape of the grassland (MPAR), humus content in the soil, soil structure and the landscape factor total dry grassland area within a 1 × 1 km^2^ area was calculated for total species richness. All multivariate models are listed in Table 4.

## 4. Discussion

Pooling the three pitfall samples from each site limits the statistical power of our analysis, but we believe the data are relevant for illustrating interesting trends concerning local biodiversity of Collembola populations on fragmented dry grassland sites. For the dry grassland patches sampled, the springtails fauna was highly diverse, consisting of about 20% of the Austrian fauna and many specialised dry grassland species that can be found only in these grasslands [16,49]. The occurrence of dry grassland species in all sites and the discovery of 6 new Collembola species for the Austrian fauna (see a detailed description in [49]) underlines the conservation value of these small grassland patches at local, regional and national level. Collembola are not high priority for conservation, but they can contribute with a high local biodiversity and high endemic richness to the conservation discussion (see [66] for Collembola diversity and endemism in the Pyrenees). Our findings support [67], who also found that grasslands are important habitats for soil biodiversity conservation.

As hypothesized, we found that the dry grassland vegetation, soil temperature and soil texture were the most important predictors for dry grassland Collembola community composition; all can be related to habitat quality for the Collembola, and that a habitat quality gradient is separating the sites along one ordination axis. Only patches 37, 38 and 39 were clearly separated from the main cluster that shows a clear gradient of the sites. Their differentiation from the main cluster can be explained biogeographically and because of very specific and sandy soils at all three patches. Vegetation cover and sand content in the soil are the most important variables separating these three patches. We could only partly confirm our hypothesis that dry grassland Collembola species richness is significantly correlated with patch variables, as this was only true for the dry grassland total Collembola species richness data set, not for the dry grassland specialist species richness.

The significant correlation between dry grassland plants and the dry grassland specialist species richness is probably because of similar habitat requirements and a direct result of interactions between the two taxonomic groups. Plant species richness might be a micro-habitat diversity measure and the soil variables can be related to habitat quality. Greenslade [68] also found at 22 native grassland sites in Australia correlations between the grassland plant communities and Collembola communities and abundances. Sabais [69] found that plant species richness was an important factor for the abundance and diversity of Collembola in temperate grasslands. Soil type, soil temperature and vegetation type also influence the Collembola communities [8,9,10,11,12,14,15], but in most of these studies, soil living Collembola (endogeic) were investigated.

On the contrary to our hypothesis, we found a significant influence of the landscape variable distance to the nearest large dry grassland and dry grassland area within 1 × 1 km^2^ underline the importance of landscape structure for Collembola diversity. In the same landscape and geographical region Querner et al. [27] found that the species richness of surface active Collembola collected in oil seed rape fields was positively correlated to landscape parameters landscape diversity, habitat richness, isolation of open habitats and area of oilseed rape fields at small to medium (250–1000 m) and at larger (1000–1750 m) spatial scales. These results suggest that parameters associated with landscape diversity can be good predictors of Collembola diversity at small scales probably due to active migration from bordering hedges, forests or grasslands, and, at larger scales, possibly due to passive wind dispersal.

We further hypothesized that the size of grassland patches is positively correlated with total species richness. We did not find correlations of the historic or current patch size with the total species richness, or any of the categories of the surface active Collembola. This shows that even small patches have a high relevance for biodiversity conservation of springtails. Because of their small body size (0.02–2 mm) and large populations in grassland soils (10^4^–10^5^ individuals m^−2^; [2]), even the smallest patches investigated (0.01 ha) contain a large number of species.

If patch size is not influencing the Collembola of the investigated sites, are the springtails then influenced by the fragmentation of the patches? Shape of the patch (MAPR) was found to be a relevant factor and could show that edge effects are influencing Collembola. Further, the distance to the nearest large dry grassland was also found to influence the springtails richness; this suggests that the landscape configuration seems to effect even small arthropods like Collembola. We present two possible explanations: Firstly, a shorter distance to the nearest large grassland can reflect a historic connection of the patch. Secondly, if patch and large grasslands are in close proximity, they will probably have similar soil properties and dry habitat conditions, resulting in large number of dry grassland species. Another (untested) is the passive transportation of Collembola by wind. If the distance between the small grassland patch and the nearest large dry grassland area is short, wind dispersal might be a more likely and frequent event. Wind dispersal for springtails was first described by [70] in an experiment collecting animals by aeroplane at a height of 3000 m. Passive dispersal by wind is a common phenomenon and can transport Collembola over long distances [71,72,73,74]. We assume that the large dry grassland areas also contain a large number of habitat-specific species, with a small number of individuals regularly dispersed in the landscape.

In recent years, some other studies investigated landscape scale effects of habitat fragmentation on soil animals like Collembola, but it is not yet clear how much these small and soil living organisms are directly and indirectly affected by habitat fragmentation and the landscape composition. Effects of forest fragmentation on soil living springtails communities were studied by Chust et al. [19,20,21] in fragmented Pyrenean forest sites, and a negative relationship between landscape heterogeneity and richness of the endemic species was found, indicating that landscape fragmentation is a potential threat to the endemic component of soil assemblages. Collembola communities were investigated by Martins da Silva et al. [22] along a gradient of forest fragmentation in different agro-forestry systems in Europe and found that environmental factors at the patch scale explained the larger part of community changes. Landscape variables were not significantly different across all study sites but an increase in forest habitat and proximity of forest patches showed an indirect influence on local community, by influencing microhabitat heterogeneity at lower spatial scales.

## 5. Conclusions

We conclude that habitat parameters (dry grassland plant species, soil temperature and soil composition) are good predictors for the dry grassland Collembola richness and community composition, but that the size of the patches is not a limiting factor. In addition, landscape scale effects might be more implication then expected in soil animal ecology.

## Figures and Tables

**Table 1:** Species list of the Collembola found on 50 dry grassland sites in Eastern Austria.

Species	Category *	Site Occupancy, %
**Order Poduromorpha** *Hypogastrura assimilis*	3	24
*Ceratophysella bengtssoni*	3	18
*Ceratophysella sigillata*	3	4
*Ceratophysella succinea*	3	36
*Schoettella ununguiculata*	^§^	12
*Choreutinula inermis*	^§^	2
*Xenylla grisea*	3	6
*Xenylla maritima*	^§^	4
*Brachystomella parvula*	3	8
*Microgastrura duodecimoculata*	3	2
*Pseudachorutes dubius*	^§^	6
*Pseudachorutes parvulus*	3	72
*Pseudachorutes subcrassus*	^§^	4
*Pseudachorutes palmiensis*	^§^	8
*Deutonura conjuncta*	^§^	20
*Neanura alba*	^§^	4
*Neanura muscorum*	^§^	2
*Odontella empodialis*	^§^	2
*Protaphorura armata*	3	8
*Protaphorura gisini*	^§^	6
*Protaphorura subfimata*	1	2
*Metaphorura affinis*	^§^	2
*Metaphorura riozoi*	1	2
**Order Entomobryomorpha** *Folsomia quadrioculata*	3	18
*Folsomides angularis*	3	4
*Proisotoma crassicauda*	^§^	2
*Proisotoma minuta*	3	6
*Proisotoma* sp.	^§^	48
*Isotoma olivacea*	3	26
*Isotoma* sp.	^§^	16
*Isotoma notabilis*	3	54
*Isotoma viridis*	3	90
*Entomobrya atrocincta*	2	4
*Entomobrya multifasciata*	1	80
*Entomobrya handschini*	1	80
*Entomobrya marginata*	2	8
*Entomobrya quinquelineata*	2	2
*Entomobrya* sp. ^1^	^§^	42
*Entomobrya* sp. ^2^	^§^	14
*Orchesella cincta*	3	98
*Orchesella flavescens*	^§^	2
*Orchesella multifasciata*	^§^	14
*Orchesella spectabilis*	1	2
*Orchesella pannonica*	1	2
*Orchesella villosa*	3	2
*Orchesella taurica*	^§^	18
*Orchesella xerothermica*	2	2
*Seira dollfusi*	2	14
*Heteromurus major*	2	60
*Lepidocyrtus cyaneus*	3	96
*Lepidocyrtus languinosus*	3	68
*Lepidocyrtus lignorum*	3	78
*Lepidocyrtus paradoxus*	3	82
*Lepidocyrtus nigrescens*	1	37
*Pseudosinella alba*	^§^	14
*Pseudosinella decipiens*	3	24
*Pseudosinella octopunctata*	^§^	6
*Pseudosinella sexoculata*	3	4
*Pseudosinella imparipunctata*	3	2
*Pseudosinella petterseni*	3	6
*Willowsia buski*	3	2
*Willowsia nigromaculata*	3	2
*Tomocerus flavescens*	^§^	34
*Tomocerus vulgaris*	3	2
*Cyphoderus albinos*	^§^	14
*Cyphoderus bidenticulatus*	2	2
**Order Symphypleona** *Sphaeridia pumilis*	3	14
*Sminthurinus elegans*	2	58
*Sminthurinus niger*	3	38
*Sminthurinus aureus*	3	38
*Sminthurinus bimaculatus*	2	2
*Sminthurinus* sp.	^§^	2
*Stenognathellus denisi*	2	2
*Dicyrtoma fusca*	^§^	4
*Sminthurus multipunctatus*	1	60
*Sminthurus viridis*	3	92
*Caprainea marginata*	3	2
*Sminthurus maculatus*	1	4
*Bourletiella viridescens*	3	2
*Deuterosminthurus bicinctus*	3	2
*Deuterosminthurus sulphureus sulphureus*	2	6
*Deuterosminthurus pallipes*	1	70
*Deuterosminthurus* sp.	^§^	2
*Heterosminthurus bilineatus*	3	2
*Heterosminthurus insignis*	3	2
*Fasciosminthurus* sp.	^§^	2

* 1 = dry grassland specialist, 2 = dry grassland generalist, 3 = generalist. ^§^ cannot be allocated to a category. Site occupancy: % of the studied patches, where the species was found.

**Table 2:** Total abundance, no. of individuals and species richness of surface-active Collembola on 50 grassland patches in eastern Austria.

Site	Area, m^2^	Total Abundance, No. Individuals	Total	Number of Species			
				Dry Grassland Specialists	Grassland Species	Total Grassland Species	Generalists
01	2158	371	19	4	2	6	11
02	3503	681	23	5	3	8	9
03	2579	1025	19	3	2	5	11
04	9202	1400	19	3	2	5	13
05	97,271	493	18	3	3	6	11
06	13,338	834	20	3	2	5	9
07	7130	2013	16	3	1	4	8
08	13,902	1614	15	2	1	3	9
09	1638	556	23	3	1	4	15
10	2905	472	19	4	2	6	11
11	870	530	17	1	2	3	11
12	5588	1615	21	1	2	3	12
13	3117	777	21	4	2	6	13
14	1713	516	17	5	3	8	9
15	4699	1200	22	5	1	6	11
16	6737	549	16	3	1	4	11
17	878	624	16	5	1	6	10
18	63,538	5690	18	5	3	8	8
19	7816	990	16	4	0	4	10
20	7214	206	16	3	1	4	10
21	28,342	424	19	4	3	7	9
22	7934	1112	19	4	2	6	9
23	1556	786	24	4	2	6	11
24	60,701	1064	17	3	1	4	10
25	3808	1200	15	2	0	2	10
26	1257	1023	21	4	1	5	10
27	5224	578	15	4	2	6	6
28	391	865	18	3	1	4	10
29	2016	3637	18	4	0	4	12
30	1155	1321	23	4	3	7	10
31	13,215	2463	19	3	1	4	10
32	23,492	937	18	3	4	7	8
33	89,674	1036	17	4	0	4	10
34	4297	394	17	5	2	7	10
35	11,777	980	23	4	2	6	14
36	1842	274	13	3	3	6	7
37	6073	1569	20	2	1	3	9
38	450	1917	20	2	1	3	12
39	5368	204	14	1	0	1	10
40	7078	310	20	5	2	7	10
41	1027	487	21	3	2	5	11
42	21,454	5576	17	3	1	4	12
43	3154	1493	17	1	1	2	10
44	53,203	496	16	3	2	5	9
45	1043	704	17	3	1	4	12
46	487	659	20	3	4	7	11
47	1076	676	16	3	1	4	11
48	22,972	328	18	5	1	6	11
49	7189	259	14	2	0	2	11
50	381	1437	21	5	1	6	10

**Table 3:** Independent variables influencing springtail species richness in 50 dry grassland patches in eastern Austria.

Response Parameter	Independent Variable	r	*P*
Dry grassland specialist species richness	Dry grassland plant species richness	0.48	<0.01
	Soil gravel content	0.353	<0.05
	Soil temperature	0.282	<0.05
	Distance to the nearest source area, mainland	0.379	<0.01
Generalist species richness	Total plant species richness	0.35	<0.05

**Table 4:** Results from the significant multivariate regression models calculated with backwards elimination for several species richness measures of Collembola found in 50 dry grassland sites in Eastern Austria.

Species Group/Background Variable	Unstandardized		Standardized		
	B	Standardised Beta	β	Student’s *t*	P
*Dry grassland specialist species richness*	−0.1539	0.8649		−0.1779	0.8596
All plant species richness	0.0527	0.0123	0.5015	4.2738	0.0001
MPAR	0.0005	0.0002	0.2656	2.2611	0.0286
Soil temperature	0.0769	0.0275	0.3167	2.7980	0.0075
Distance to nearest large dry grassland	0.0001	0.0000	−0.2777	−2.4736	0.0172
*Grassland species richness*	−1.6670	1.2521		−1.3314	0.1896
All plant species richness	0.0818	0.0189	0.5244	4.3217	0.0001
MPAR	0.0006	0.0003	0.2432	1.9881	0.0528
Soil temperature	0.1433	0.0429	0.3977	3.3355	0.0017
*Generalist species richness*	12.7616	0.7648		16.6866	0.0000
All plant species richness	−0.0924	0.0218	−0.6119	−4.2367	0.0001
Humus	0.1549	0.0454	0.4930	3.4136	0.0013
*Total species richness*	21.7679	2.0429		10.6552	0.0000
MPAR	0.0012	0.0005	0.2899	2.2315	0.0309
Humus	0.3596	0.1171	0.7137	3.0712	0.0037
Gravel	−0.0453	0.0189	−0.3421	−2.1315	0.0388
Clay	−0.2259	0.0654	−1.0045	−3.4558	0.0012
